# Reduced Light Response of Neuronal Firing Activity in the Suprachiasmatic Nucleus and Optic Nerve of *Cryptochrome*-Deficient Mice

**DOI:** 10.1371/journal.pone.0028726

**Published:** 2011-12-21

**Authors:** Takahiro J. Nakamura, Shizufumi Ebihara, Kazuyuki Shinohara

**Affiliations:** 1 Division of Neurobiology and Behavior, Department of Translational Medical Sciences, Graduate School of Biomedical Sciences, Nagasaki University, Nagasaki, Japan; 2 Division of Biomodeling, Graduate School of Bioagricultural Sciences, Nagoya University, Nagoya, Japan; 3 Faculty of Pharmaceutical Sciences, Teikyo Heisei University, Ichihara, Chiba, Japan; National Cancer Institute, United States of America

## Abstract

To examine roles of the *Cryptochromes* (*Cry1* and *Cry2*) in mammalian circadian photoreception, we recorded single-unit neuronal firing activity in the suprachiasmatic nucleus (SCN), a primary circadian oscillator, and optic nerve fibers *in vivo* after retinal illumination in anesthetized *Cry1* and *Cry2* double-knockout (*Cry*-deficient) mice. In wild-type mice, most SCN neurons increased their firing frequency in response to retinal illumination at night, whereas only 17% of SCN neurons responded during the daytime. However, 40% of SCN neurons responded to light during the daytime, and 31% of SCN neurons responded at night in *Cry*-deficient mice. The magnitude of the photic response in SCN neurons at night was significantly lower (1.3-fold of spontaneous firing) in *Cry*-deficient mice than in wild-type mice (4.0-fold of spontaneous firing). In the optic nerve near the SCN, no difference in the proportion of light-responsive fibers was observed between daytime and nighttime in both genotypes. However, the response magnitude in the light-activated fibers (ON fibers) was high during the nighttime and low during the daytime in wild-type mice, whereas this day–night difference was not observed in *Cry*-deficient mice. In addition, we observed day–night differences in the spontaneous firing rates in the SCN in both genotypes and in the fibers of wild-type, but not *Cry*-deficient mice. We conclude that the low photo response in the SCN of *Cry*-deficient mice is caused by a circadian gating defect in the retina, suggesting that *Cryptochromes* are required for appropriate temporal photoreception in mammals.

## Introduction

Circadian rhythms are oscillations with daily periodicities in physiological and behavioral functions of organisms. In mammals, the central circadian oscillator is located in the suprachiasmatic nucleus (SCN) of the ventral hypothalamus [1]. The rhythms are generated by a cell-autonomous circadian oscillator that is synchronized with the environment by light through the retinohypothalamic tract; hence, light synchronizes the behavior of the organism with the daily 24-hr light–dark (LD) cycle [2].

The mammalian retina mediates several nonvisual light-responsive functions, including circadian photoreception [3], acute suppression of locomotor activity by light (masking) [4], photic suppression of pineal melatonin synthesis [5], and pupillary light responses [6]. Candidate photoreceptors for these functions include opsins, such as classical rod and cone opsins and the novel photopigment melanopsin [7], and the blue-light photoreceptive pigments *Cryptochromes* [8]. In the *rd* (retinal degeneration) mouse, a mutation in a rod-specific gene [9] leads to the rapid degeneration of rod cells. Rod degeneration is complete after 2 months whereas the secondary degeneration of cones is much slower [10]. While these *rd/rd* mice lack the photopigments for vision, they retain robust nonvisual responses to light [3], [4], [5], [6], [11], indicating the involvement of other nonclassical photoreceptors. Melanopsin knockout mice (*Opn4^−/−^*) exhibit a somewhat reduced sensitivity to circadian phase shifting [12], [13] and masking [12] but display normal entrainment [12], [13] and nearly normal pupillary light reflex [14], [15] and gene induction by light in the SCN [13]. Indeed, *rd/rd Opn4^−/−^* mice displayed severe defects in all tested photoreceptive tasks [15], [16].

*Cryptochromes* are folate- and flavin-based members of the photolyase family of photopigments that are necessary for normal circadian phase shifting in *Arabidopsis* and *Drosophila* [17]. Mammals have two *Cryptochrome* family members, which are expressed in the inner retina [17], [18] as well as many other tissues. Mice lacking *Cryptochromes* (*mCry1^−/−^ mCry2^−/−^*) display severe defects in gene induction in the SCN [19], [20], but retain normal pupillary light reflex [21] and masking [22]. Photoresponsiveness is markedly depressed in *Rd/rd mCry1^−/−^ mCry2^−/−^* mice, as measured by masking, pupillary light reflex, and light-induced immediate-early gene expression in the SCN [19], [21], [23]. These studies indicate that both melanopsin and *Cryptochromes* contribute to nonvisual photoresponses and play important roles in these processes.

Because *Cryptochromes* also have a central role for these proteins in the molecular clock mechanism [20], [24], *mCry1^−/−^ mCry2^−/−^* mice show arrhythmic behavior in constant darkness (DD) [19], [24] and cannot be assayed for circadian phase shifting. Therefore, in the present study, we performed extracellular single unit recordings of the neuronal firing activity in the SCN and the optic nerve of anesthetized *mCry1^−/−^ mCry2^−/−^* mice in response to retinal illumination. Several rodent studies have shown that the photic responses of the electrical activities in the SCN are closely correlated with the photic entrainment properties in the locomotor activity rhythms [25], [26], [27]. The magnitude of the photic responses of electrical activities in the SCN depends on both the circadian phase [27] and the light intensity [25], [26], [27] in rats and hamsters. Recently, we successfully recorded the photic response in the firing of mouse SCN *in vivo*, which corresponds to the properties of photic entrainment in the locomotor activity of mice [28]. In the present study, to assay circadian photoreceptions in *mCry1^−/−^ mCry2^−/−^* mice, we compared the light response of neuronal firing activity in the SCN and the optic nerve during the daytime and the nighttime.

## Results

For recordings in the SCN and optic fibers, we used 46 wild-type, 69 *mCry1^−/−^ mCry2^−/−^* mice. We carried out a single recording per animal in which 16 neurons in wild-type mice and 26 neurons in *mCry1^−/−^ mCry2^−/−^* mice were recorded in the SCN. The rest of the animals were used for optic nerve fiber recordings.

### Temporal differences in spontaneous neuronal firing activity in the SCN and optic nerve fibers

**Figure 1:**
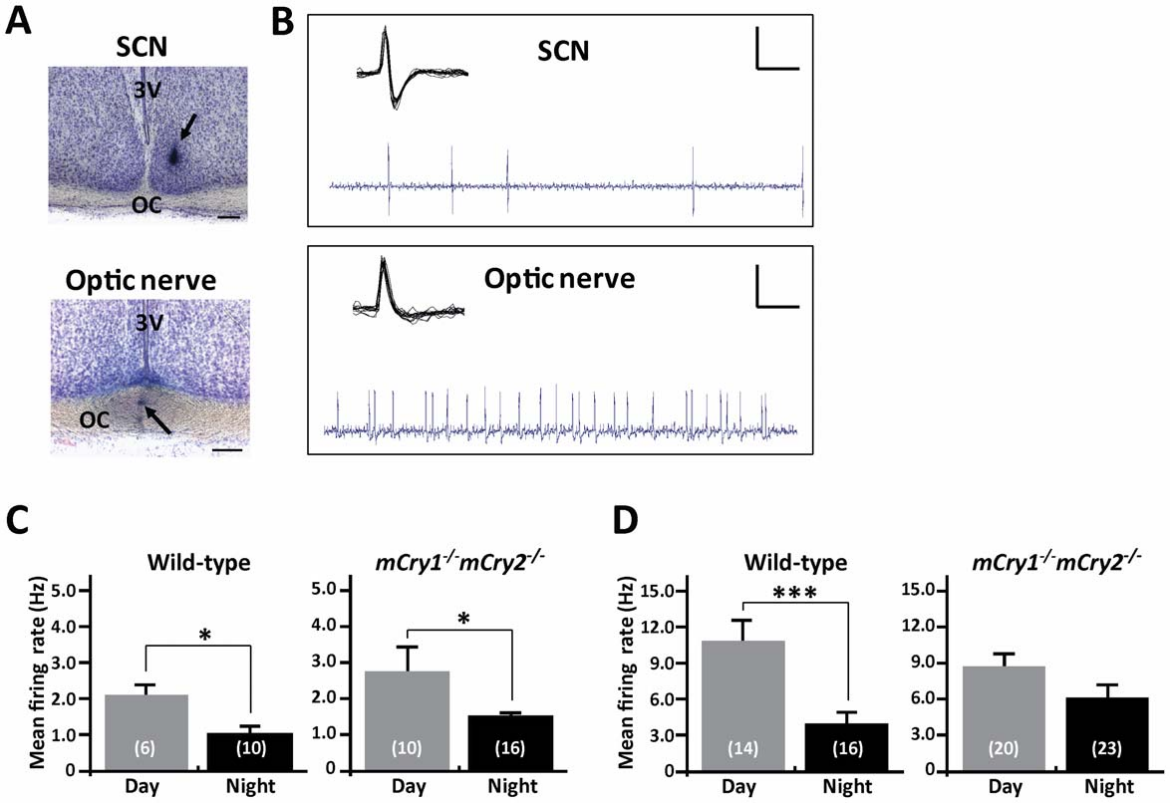
Temporal differences in spontaneous neuronal firing activity in the SCN and optic nerve of wild-type and *mCry1^−/−^ mCry2^−/−^* mice. **A**, Representative photographs show the recording sites of the SCN (top panel) or optic nerve fibers (bottom panel). Arrow-heads indicate the typical recording positions. 3V, 3rd ventricle; OC, optic chiasm. Scale bars indicate 200 µm. **B**, Representative “*light-activated*” neuronal firings recorded in the SCN neuron and the optic nerve fibers of mice are shown. Each inset shows the characteristic waveform of the spike. Vertical calibration bars, 0.5 mV; horizontal calibration, 0.1 sec (oscilloscope trace) and 10 msec (inset). **C** and **D**, Day–night differences in the frequencies of spontaneous neuronal firing activity in the SCN (C) and optic nerve (D) of wild-type and *mCry1^−/−^ mCry2^−/−^* mice. Histograms depict data as mean ± SEM; the number of animals is shown in parentheses. * *P*<0.05, *** *P*<0.001, Student's *t*-test.

Representative photographs and oscilloscope traces for the single unit recordings of the neuronal firing activity in the SCN and the optic nerve fibers of mice are shown in Figure 1A and B. We clearly distinguished spikes between the SCN and the optic nerve by means of the spike form and additionally confirmed the recording site using the histological method after the recording. We first measured the baseline of spontaneous firing activity in the SCN and optic nerve fibers of mice during the daytime (Zeitgeber time [ZT] 4–8) and the nighttime (ZT 14–16). We collected the baseline firing frequency (Hz) for 60 sec without retinal illumination. In the SCN, both wild-type and *mCry1^−/−^ mCry2^−/−^* mice displayed a day–night variation in spontaneous firing activity (2.03±0.37 during the daytime vs. 1.09±0.21 during the nighttime in wild-type mice and 2.83±0.67 during the daytime vs. 1.54±0.17 during the nighttime in *mCry1^−/−^ mCry2^−/−^*; *P<0.05* for both genotypes, Student's *t*-test; Fig. 1C). In the optic nerve, wild-type mice showed a distinct day–night change (10.99±1.53 during the daytime vs. 4.13±0.72 during the nighttime; *P<0.001*, Student's *t*-test; Fig. 1D), whereas *mCry1^−/−^mCry2^−/−^* mice did not exhibit a day–night difference in spontaneous firing rate (8.72±0.12 during the daytime vs. 6.22±1.19 during the nighttime; Fig. 1D).

### SCN light responsiveness

**Figure 2:**
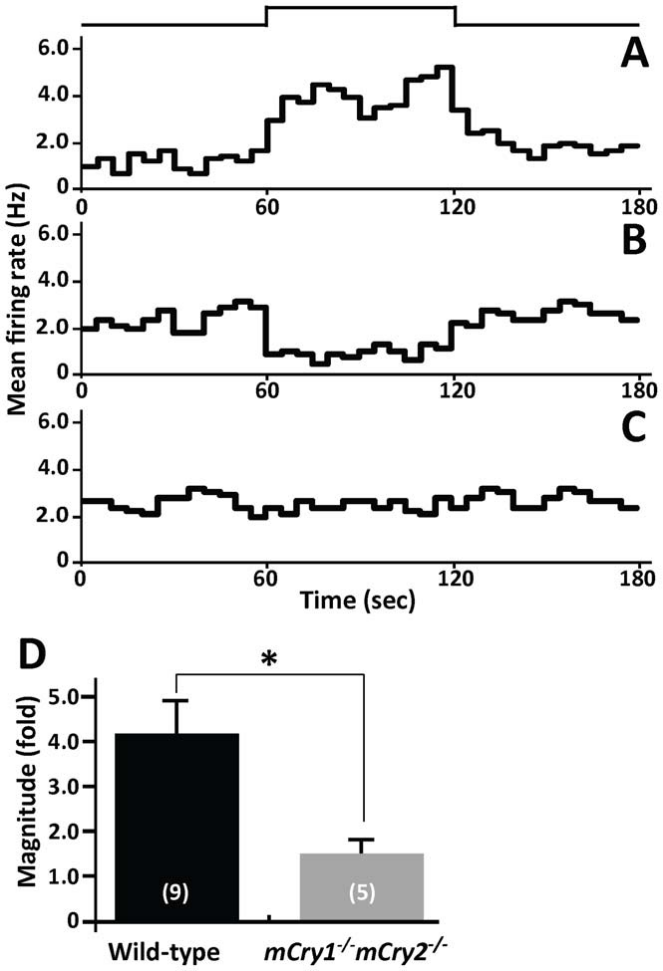
Reduced light response of neuronal firing activity in the SCN of *mCry1^−/−^ mCry2^−/−^* mice. **A**, **B**, and **C**, Peristimulus time histograms of three response patterns in firing activity are shown: (A) light-activated, (B) light-suppressed, and (C) unresponsive. The mean firing rate per second (Hz) is plotted every 5 min in the histogram. The light intensity was 1.0×10^15^ photons·cm^−2^·s^−1^ for 60 sec. The timing of the light pulse is indicated in the step diagram above the records. **D**, The magnitude of change in the discharge rate for retinal illumination recorded in the SCN. Histograms depict data as mean ± SEM; the number of animals is shown in parentheses. * *P*<0.05, Student's *t*-test.

The spontaneously firing SCN neurons of mice showed three different types of responses to light [28]. The recordings consisted of before, during, and after retinal stimulation and each stage was applied for 60 sec. The first type of response was *light-activated*, in which the firing frequency increased during light exposure (Fig. 2A), the second type of response was *light-suppressed*, in which the firing rate decreased during light exposure (Fig. 2B), and the third type of response was unresponsive in which changes in the firing rate during light exposure were less than 10% of the basal firing rate (Fig. 2C).

We compared the differences in the populations of the three types of responses in the SCN during the daytime and nighttime in wild-type and *mCry1^−/−^ mCry2^−/−^* mice (Table 1). In wild-type mice, the spontaneous activities of six SCN neurons were recorded in the daytime and one (17%) of the SCN neurons was light-activated whereas the remaining neurons (83%) were unresponsive. During the nighttime, 10 SCN neurons were recorded in wild-type mice and nine (90%) of them were light-activated and one neuron (10%) was light-suppressed. Statistical analysis revealed that a day–night difference in the populations of response types in SCN neurons of wild-type mice (*P*<0.001, Dunn's test). In contrast, among 26 SCN neurons that were recorded in *mCry1^−/−^ mCry2^−/−^* mice, 16 were recorded during the nighttime. In the nighttime recording, five neurons (31%) were light-activated and 11 neurons (69%) were unresponsive. Among the 10 neurons recorded during the daytime, one neuron (10%) was light-activated, three neurons (30%) were light-suppressed, and the remaining neurons (60%) were unresponsive. A day–night variation was not observed in the populations of response types in the SCN neurons of *mCry1^−/−^ mCry2^−/−^* mice.

**Table 1:** Temporal difference in frequency of the response types in the SCN of wild-type and *mCry1^−/−^mCry2^−/−^* mice.

Genotype	Recording time	Response		Unresponsive
		Activated	Suppressed	
Wild-type	Daytime (n = 6)	1 (17%)	0 (0%)	5 (83%)
		1.00/4.00/0.90	n.d.	2.24/2.22/2.20
	Nighttime (n = 10)*	9 (90%)	1 (10%)	0 (0%)
		0.98/3.00/0.93	2.10/1.30/2.10	n.d.
*mCry1^−/−^mCry2^−/−^*	Daytime (n = 10)	1 (10%)	3 (30%)	6 (60%)
		1.45/1.50/1.25	1.60/1.17/1.57	3.67/3.63/3.67
	Nighttime (n = 16)	5 (31%)	0 (0%)	11 (69%)
		1.48/2.46/2.00	n.d.	1.56/1.63/1.65

The actual number and proportion (%) of responding cells in mouse SCN during daytime and nighttime are shown. The mean firing rates of each responding cell are exhibited in the bottom of each column described as before/during/after light stimulation.

*P<0.001, when compared with daytime group of wild-type mice and *mCry1^−/−^mCry2^−/−^* mice (Dunn's test). n.d.; not determined.

We next examined the magnitude of change in neuronal firing activities by retinal illumination recorded in the SCN of wild-type and *mCry1^−/−^ mCry2^−/−^* mice (Fig. 2D). Because few light-responsive neurons were observed during the daytime, the recording was carried out only in light-activated neurons during the nighttime. We collected the mean firing rate at each stage of light stimulation: before (60 sec), during (60 sec), and after (60 sec) light stimulations. The magnitude of neuronal light response was calculated by using the following equation:
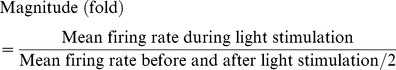
In the SCN, wild-type mice showed a magnitude of 4.01±0.79-fold whereas *mCry1^−/−^ mCry2^−/−^* mice showed 1.35±0.15-fold. The magnitude of the neuronal light response in the SCN of *mCry1^−/−^ mCry2^−/−^* mice during the nighttime was significantly lower than in the wild-type mice (*P*<0.05, Student's *t*-test).

### Optic nerve fiber light responsiveness

**Figure 3:**
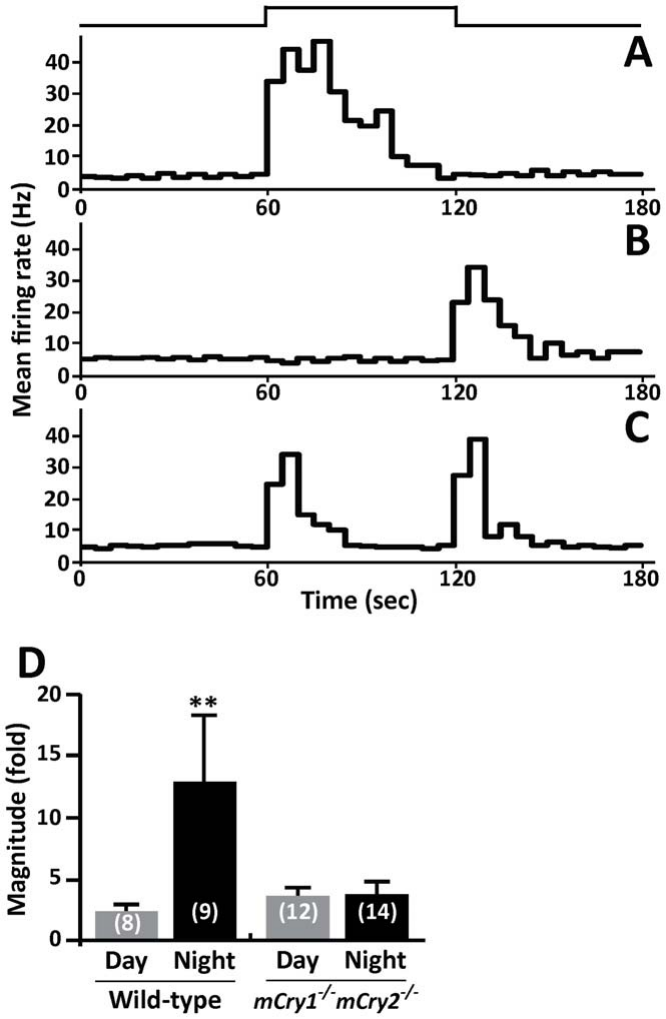
Reduced light response of neuronal firing activity in the optic nerve fibers of *mCry1^−/−^ mCry2^−/−^* mice. **A**, **B**, and **C**, Peristimulus time histograms of three distinct response patterns were recorded: ON fibers, which respond to light onset (A), OFF fibers, which respond to light offset (B), and ON/OFF fibers, which respond to both light onset and offset (C). The mean firing rate per second (Hz) is plotted every 5 min in the histogram. Light intensity was 1.0×10^15^ photons·cm^−2^·s^−1^ for 60 sec. The timing of the light pulse is indicated in the step diagram above the records. **D**, The magnitude of change in the discharge rate for retinal illumination recorded in the ON fiber. Histograms depict data as mean ± SEM; the number of animals is shown in parentheses. **P<0.01 vs. others, Tukey's test.

To determine whether the reduced light response in the SCN of *mCry1^−/−^ mCry2^−/−^* mice was caused by retinal defects, we examined the response to retinal illuminations in firing activity in the optic nerve, which the neural light information pathway to the SCN. The recordings and light stimulations were performed using the same procedure as for the SCN recording. We recorded three classes of light responses in optic nerve fibers, which is consistent with Hartline et al. [29] and other reports [30], [31]. The first type was the ON fiber that discharges vigorously when the retina is illuminated (Fig. 3A), the second was the OFF fiber that discharges vigorously when the light is turned off (Fig. 3B), and the third was the ON/OFF fiber that responds to both the onset and the termination of light (Fig. 3C).

We compared the differences in the populations of the three fibers in the optic nerve during the daytime and the nighttime in wild-type and *mCry1^−/−^ mCry2^−/−^* mice (Table 2). In wild-type mice, the spontaneous activities of 14 optic nerve fibers were recorded in the daytime and eight (57%) were ON fibers, four (29%) were OFF fibers, and two (14%) were ON/OFF fibers. During the nighttime, 16 fibers were recorded in wild-type mice and nine (56%) were ON fibers and seven (44%) were OFF fibers. In *mCry1^−/−^ mCry2^−/−^* mice, 20 fibers were recorded in the optic nerve during the daytime and twelve (60%) were ON fibers, six (30%) were OFF fibers, and the remaining (10%) were ON/OFF fibers. During the nighttime, 23 fibers were recorded and 14 (61%) were ON fibers, seven (30%) were OFF fibers, and the remaining (9%) were ON/OFF fibers. No day–night variation was observed in the populations of the three classes of fibers in the optic nerve of wild-type or *mCry1^−/−^ mCry2^−/−^* mice.

**Table 2:** Temporal difference in frequency of the ON, OFF, and ON/OFF fibers of wild-type and *mCry1^−/−^mCry2^−/−^* mice.

Genotype	Recording time	ON fiber	OFF fiber	ON/OFF fiber
Wild-type	Daytime (n = 14)	8 (57%)	4 (29%)	2 (14%)
		12.89/25.61/11.58	7.20/1.08/9.08	11.00/17.60/20.70
	Nighttime (n = 16)	9 (56%)	7 (44%)	0 (0%)
		3.91/22.09/4.46	4.43/0.87/3.51	n.d.
*mCry1^−/−^mCry2^−/−^*	Daytime (n = 20)	12 (60%)	6 (30%)	2 (10%)
		7.52/15.56/3.03	11.18/4.68/9.90	8.50/20.75/17.33
	Nighttime (n = 23)	14 (61%)	7 (30%)	2 (9%)
		6.53/14.23/9.09	5.61/2.67/6.06	6.20/13.00/20.15

The actual number and proportion (%) of types of the optic fiber during daytime and nighttime are shown. The mean firing rates of each responding cell are exhibited in the bottom of each column described as before/during/after light stimulation. n.d.; not determined.

We also examined the magnitude of the neuronal light response recorded in the optic nerve fibers of wild-type and *mCry1^−/−^ mCry2^−/−^* mice (Fig. 3D). The recordings were carried out only in ON fibers during the daytime and the nighttime because OFF and ON/OFF fibers exhibit a specific response to retinal illuminations and thus these fibers could not be assayed in the present study. The magnitudes of the neuronal light response during the daytime and nighttime recordings were 2.64±0.60-fold and 13.36±4.41-fold, respectively, in the ON fibers of wild-type mice. In contrast, the magnitude of the neuronal light response during the daytime and nighttime recordings were 3.23±0.57-fold and 3.02±0.82 fold, respectively, in *mCry1^−/−^ mCry2^−/−^* mice. The magnitude of the neuronal light response in the ON fibers of wild-type mice during the nighttime was significantly higher than both the daytime value in wild-type mice and the day and night values in *mCry1^−/−^ mCry2^−/−^* mice (*P*<0.01; Tukey's test). Thus, a day–night difference was observed in the magnitude of the neuronal light response in the ON fibers of wild-type mice but not in *mCry1^−/−^ mCry2^−/−^* mice.

## Discussion

In the present study, we investigated the effect of the loss of *Cryptochromes* on the light response of neuronal firing activity in the SCN. We revealed that *mCry1^−/−^ mCry2^−/−^* mice have a reduced firing activity response to retinal illumination in nighttime recordings and did not exhibit a day–night variation in frequency of the response types in the SCN. We also determined that *mCry1^−/−^ mCry2^−/−^* mice have decreased optic nerve fiber photosensitivity of optic nerve fibers during the night. These results suggest that *Cryptochromes* play a key role in the sensitivity of circadian photoreception in mammals.

Our previous study demonstrated that the photo response of firing activity in the mouse SCN had phase-dependent manner and showed a light-intensity relationship [28]. These data indicate that the electrophysiological properties of mice SCN are similar to those of rats and hamsters [25], [26], [27] and may correspond to properties of the light-induced phase shifting in locomotor activity of mice. In the present results, the SCN of wild-type mice showed a day–night difference in the populations of light responsive neurons. On the other hand, a day–night variation was not observed in the proportion of response types in the SCN of *mCry1^−/−^ mCry2^−/−^* mice. The magnitudes of the neuronal light responses in the SCN of *mCry1^−/−^ mCry2^−/−^* mice were significantly lower than those of wild-type mice during the night. *mCry1^−/−^ mCry2^−/−^* mice displayed a severe defect in *c-fos* induction in the SCN [19], [20] and no day–night difference in *c-fos* induction in the SCN [32]. These data are consistent with the present results, suggesting that circadian photoreception sensitivity is reduced in the SCN of *mCry1^−/−^ mCry2^−/−^* mice.

Because *Cryptochromes* have a transcriptional regulatory function in the molecular clock mechanism, *mCry1^−/−^ mCry2^−/−^* mice do not exhibit circadian rhythms of locomotor activity in DD [19], [24], suggesting that the SCN lacks to the central clock function in *mCry1^−/−^ mCry2^−/−^* mice. Although it appears that the loss of the day–night difference in the photo response in the SCN of these animals causes the lack of SCN function, we consider this model unlikely because some evidence for partial clock function in the absence of *Cryptochromes* has been reported. For example, anticipatory wheel-running activity, where mice exhibit increased locomotor activity in the hours just prior to lights off, has been reported in *mCry1^−/−^ mCry2^−/−^* mice [20], [22]. Under normal LD conditions, a single peaks in circadian multiunit electrical activities were detected in the *in vitro* SCN slices from these animals [33]. Our present results also show a temporal difference in the spontaneous neuronal of the SCN in *mCry1^−/−^ mCry2^−/−^* mice. These data suggest that *mCry1^−/−^ mCry2^−/−^* mice maintained under LD conditions have normal clock functions of the SCN through the duration of one circadian cycle. Because we recorded the neuronal firing activity of these animals under LD conditions, the SCN showed normal clock functions in *Cry*-deficient mice in the present study.

Our data demonstrate that the light response of ON fibers also exhibited a day-night difference in wild-type mice, although the day–night variation was not observed in the populations of optic nerve fibers in wild-type or *mCry1^−/−^ mCry2^−/−^* mice. Circadian rhythms have been detected in numerous invertebrate visual systems but are not unique to them [34]. A circadian clock in the brain of *Limulus* transmits efferent optic nerve activity to the lateral eyes and increases their sensitivity at night [35] enabling them to see nearly as well at night as they do during the day. Circadian rhythms have been also detected in functions such as photoreceptor disk shedding [36] and retina light sensitivity of the retina in rats [37] and humans [38]. Therefore, it is possible that the day–night difference in the sensitivity of ON fibers is relevant to circadian photoreceptions. The observation that the ON fibers of *mCry1^−/−^ mCry2^−/−^* mice lack these day–night variations may lead to the finding that *Cryptochromes* play a role in the retina for mammalian circadian photoreception.

The discovery of intrinsically photoresponsive retinal ganglion cells (ipRGCs) has given non-visual phototransduction an anatomical basis [39]. Berson and colleagues used retrograde dye tracing from the circadian pacemaking cells in the rat SCN to define direct retinohypothalamic-projecting ganglion cells, and patch-clamp recording showed that these cells are found to be intrinsically photosensitive whereas non-retinohypothalamic projecting retinal ganglion cells had no intrinsic photosensitivity [40]. Although we could not determine whether all recorded optic nerve fibers were ipRGCs in the present study, *Cryptochrome* mRNAs are expressed in the retinal ganglion cells of mice [8]. Melanopsin is also expressed nearly exclusively in the ∼1000 ipRGCs of the rodent retina [41]. The mammalian retina contains an intrinsic circadian clock that controls melatonin synthesis and many other retinal functions [42]. In addition, retinal ganglion cells express *Period* (*Per*) *1* and *2*, *Clock*, and *Bmal1*, as well as *Cry1* and *2*, which are core molecular components of circadian clocks and their expression is necessary for circadian rhythmicity [43]. Furthermore, studies using real-time reporting of the PER2::LUC fusion protein revealed that clock gene rhythms persist for >25 days in cultured mouse retinas [43], [44]. These data suggest that the ipRGCs of the mammalian retina contain functionally autonomous circadian clocks. In the present study, *mCry1^−/−^ mCry2^−/−^* mice did not exhibit a day–night difference in spontaneous firing rate of the optic fibers and in the magnitude of neuronal light response in ON fibers. There results indicate that the low photosensitivity in the optic fibers is caused by the loss of *Cryptochromes* in the retina.

In summary, this study provides several findings regarding the neuronal light response in the SCN and optic fibers of *Cry*-deficient mice: (1) A day–night variation in the populations of response types in SCN neurons was observed in wild-type mice but not in *mCry1^−/−^ mCry2^−/−^* mice. (2) The magnitude of the neuronal light response in the SCN of *mCry1^−/−^ mCry2^−/−^* mice during nighttime was significantly lower than in wild-type mice. (3) A day–night difference was observed in the magnitude of neuronal light response in the ON fibers of wild-type mice but not in *mCry1^−/−^ mCry2^−/−^* mice. These findings indicate that CRY deletion leads to the low photo response of the SCN and optic nerve fibers during the nighttime. In addition, we observed a day–night difference in the spontaneous firing rates in the optic fibers in wild-type mice but not in *mCry1^−/−^ mCry2^−/−^* mice, suggesting that CRY deletion also disrupts the circadian rhythms of the neural system in the retina. We conclude that the low photo response in the SCN of *Cry*-deficient mice is caused by a circadian gating defect in the retina, which suggests that *Cryptochromes* are required for appropriate temporal photoreception in mammals.

## Materials and Methods

### Animals

The *mCry1^−/−^ mCry2^−/−^* mice (originally from the colony of Dr. T. Todo [Kyoto University, Kyoto, Japan]) and wild-type mice of a similar mixed background were generated as described previously [19], [20]. Genotyping was carried out by PCR using two sets of primers that amplified the wild-type or the disrupted gene for each of the *Cryptochrome* genes. Animals were maintained under controlled air conditions (room temperature, 24±1°C, and humidity, 50%±5%) with food and water available ad libitum. Animals were housed under a LD cycle of 12 hr of light and 12 hr of darkness with a light intensity of 200–300 lux until the beginning of the experiment. All animal housing and experimental procedures were carried out in accordance with the guidelines of the Japanese Physiological Society and approved by the Institutional Animal Care and Use Committee of the Graduate School of Biomedical Sciences Nagasaki University (approval ID#: 0206090168).

### Preparation

Male *mCry1^−/−^ mCry2^−/−^* mice and wild-type mice ranging from 3 to 5 months of age were used in the experiments. The experiments were carried out during the daytime, ZT 4–8 (ZT12 is defined as the time of lights-off), and nighttime, ZT 14–16. The mice studied during the daytime were transferred to constant darkness from ZT 12 on the previous day in order to prevent light adaptation in animals and to create light conditions similar to the nighttime recording. On the day of the experiment, mice were transferred to the experimental room after their eyes were covered with blindfolds. The surgery described below, which occurred before the electrical recording, was performed under dim red light (<10 lux). The mice were anesthetized with 20% urethane solution (initial dose 2 g/kg, i.p.). Thereafter, the mice were placed in a stereotaxic instrument (Narishige, Tokyo, Japan) with the incisor bar set −2 mm below the ear bar and cranial surgery was performed. The coordinates for the SCN in mice were 0.4 mm anterior to bregma, 0.1 mm lateral to the midline, and 5.0–5.5 mm below the dural surface. The pupils of the animals were dilated 30 min before the recordings by application of 1% atropine sulfate to the cornea.

### Electrophysiological recordings

Electrophysiological experiments were performed as previously described [28]. Extracellular single unit recordings were performed with a glass micropipette electrode (10–20 MΩ) filled with 2% Chicago Sky Blue (SIGMA, St. Louis, MO) in 0.5 M NaCl. The potentials were amplified, processed through a bandpass analog filter (100 Hz–3 KHz), and fed into a personal computer with an AD converter (PCI-6024E; National Instruments, Austin, TX). The frequency of neuronal single-unit firing was counted with customized software programmed by LabVIEW (National Instruments).

When spontaneous firings were recorded, we continued recording for 10 min without illumination to ensure stability of the firing activity. The stimulation was carried out after the variation of the mean spontaneous firing rate in 60 sec settled down to a rate that was within 10% of the value of the previous minutes. An increase or decrease in the firing activity was determined for the light stimulation of 60 sec in duration by a mean frequency change of more than 10% relative to the mean firing rate for the 60 sec prior to the stimulation.

### Light stimulation

A photic stimulation pulse was applied with an assembled light source of 6 blue-green high-intensity light-emitting diodes (λmax: 500 nm, E1L51-KC0A2-02; Toyota Gosei, Kasugai, Japan) to the eye of the animal with pupils dilated. The intensity of the light stimulation at the plane of the eye was 1.0×10^15^ photons·cm^−2^·s^−1^. Light intensity was measured using a United Detector Technologies photometer (model S371; Hawthorne, CA).

### Identification of the electrode position

At the end of the recording, a small negative current (3–5 µA; 3–5 min) was passed through the microelectrode to mark the recording site. The brain was removed and fixed overnight with 4% paraformaldehyde in phosphate-buffered saline. The brain was sliced (100 µm thick) with a micro slicer (Dosaka EM, Kyoto, Japan) and slices were counterstained with cresyl violet to verify the location of the SCN or optic nerve.

### Data analysis and statistics

Differences between proportions of responsive neurons or fibers during the daytime and the nighttime in wild-type and *mCry1^−/−^ mCry2^−/−^* mice were analyzed using the non-parametric Kruskal–Wallis test followed by Dunn's post-hoc analyses. In other cases, Student's *t*-tests were used to examine the difference between two groups and one-way ANOVA with post-hoc Tukey's test was used to compare multiple groups. All results are presented as the mean ± SEM and were considered significant at *P*<0.05.
